# Mixture density networks for the indirect estimation of reference intervals

**DOI:** 10.1186/s12859-022-04846-0

**Published:** 2022-07-29

**Authors:** Tobias Hepp, Jakob Zierk, Manfred Rauh, Markus Metzler, Sarem Seitz

**Affiliations:** 1 Department of Medical Informatics, Biometry and Epidemiology, Friedrich-Alexander-Universität Erlangen-Nürnberg, Waldstraße 6, 91054 Erlangen, Germany; 2 Chair of Spatial Data Science and Statistical Learning, Georg-August-Universität Göttingen, Platz der Göttinger Sieben 3, 37073 Göttingen, Germany; 3 Department of Pediatrics and Adolescent Medicine, University Hospital Erlangen, Loschgestraße 15, 91054 Erlangen, Germany; 4 Department of Information Systems and Applied Computer Science, Otto-Friedrich-Universität Bamberg, Kapuzinerstraße 16, 96047 Bamberg, Germany

**Keywords:** Mixture density networks, Reference intervals, Latent class regression, Distributional regression

## Abstract

**Background:**

Reference intervals represent the expected range of physiological test results in a healthy population and are essential to support medical decision making. Particularly in the context of pediatric reference intervals, where recruitment regulations make prospective studies challenging to conduct, indirect estimation strategies are becoming increasingly important. Established indirect methods enable robust identification of the distribution of “healthy” samples from laboratory databases, which include unlabeled pathologic cases, but are currently severely limited when adjusting for essential patient characteristics such as age. Here, we propose the use of mixture density networks (MDN) to overcome this problem and model all parameters of the mixture distribution in a single step.

**Results:**

Estimated reference intervals from varying settings with simulated data demonstrate the ability to accurately estimate latent distributions from unlabeled data using different implementations of MDNs. Comparing the performance with alternative estimation approaches further highlights the importance of modeling the mixture component weights as a function of the input in order to avoid biased estimates for all other parameters and the resulting reference intervals. We also provide a strategy to generate partially customized starting weights to improve proper identification of the latent components. Finally, the application on real-world hemoglobin samples provides results in line with current gold standard approaches, but also suggests further investigations with respect to adequate regularization strategies in order to prevent overfitting the data.

**Conclusions:**

Mixture density networks provide a promising approach capable of extracting the distribution of healthy samples from unlabeled laboratory databases while simultaneously and explicitly estimating all parameters and component weights as non-linear functions of the covariate(s), thereby allowing the estimation of age-dependent reference intervals in a single step. Further studies on model regularization and asymmetric component distributions are warranted to consolidate our findings and expand the scope of applications.

## Background

In vitro diagnosis plays an important role in routine clinical practice and patient management with approximately 66% of medical decisions supported by the corresponding test results [1]. In order to assess whether a sample taken from a patient should be considered pathological or not, reference intervals provide supporting information about the range of values that can be expected for healthy individuals with respect to a specific analyte. Accordingly, accurate determination of the underlying distributions from which these intervals can be derived is very important and discussed in several guidelines [2, 3].

*Direct* estimation methods rely on extensive prescreening to filter out the pathological samples and use only physiological data. As a consequence, they may be considered as the most promising approach for this purpose [4], but the conduction of the required prospective studies involves considerable effort and expense. As an alternative, *indirect* methods estimate the distributions retrospectively from test results that have been already collected in the everyday clinical routine and are stored in laboratory databases [5]. This considerably lowers the burden associated with data collection, allowing smaller laboratories not only to establish own reference distributions to avoid potential transferability problems with external results but also to conduct periodical reviews from the constant flow of new data coming in. Moreover, a major advantage of indirect approaches is that the available datasets are usually much larger in size. This is particularly important with respect to analytes that are heavily influenced by (continuous) covariates and patient characteristics such as age [6]. As the dynamics of these dependency structures are particularly pronounced during the course of childhood and adolescence, collecting enough data in order to be able to appropriately reflect these patterns is additionally challenging for prospective study designs due to the strict recruitment regulations in these age groups [7, 8]. Another advantage of laboratory databases is the wide variety of available measurements of different analytes.

However, this versatility comes at a price, as there is no pre-screening step that prevents pathological samples from being included in the databases. What contributes to this problem is that a patient’s condition at the time of sample collection can have different effects on different analytes and whether or not an entry in the database should be considered pathological for a specific analyte. Comprehensive relevant information is often not available retrospectively, hence resulting in “contaminated” data with unlabeled health status. A key element of indirect estiamtion procedures is therefore the use of appropriate techniques and assumptions to avoid biased estimates for the reference distributions due to the inclusion of pathological samples in the analysis.

Several approaches have been suggested to achieve this task [9]. The major advantage of the most advanced established methods is that they work without assumptions regarding the shape of the distribution of pathological measurements [10–12], but they extract the reference distribution without being able to adjust for variations in patient characteristics such as age. For the reasons mentioned above, estimating pediatric intervals then requires a two-step approach where the data is first split into discrete age groups and the resulting intervals are subsequently interpolated to create a continuous representation (e.g. [13, 14]). To overcome this limitation, we suggested the use of conditional finite mixture models in the form of latent class distributional regression [15] by relying on generalized additive models for location scale and shape (GAMLSS) [16] to represent the latent mixture components in an expectation-maximization (EM) framework [17]. The suggested model provides an integrated approach that requires only a single fit to the data in order to account for both non-linear effects of covariates on multiple distribution parameters and unlabeled health status.

A current drawback of the approach, however, is that the proposed algorithm estimates the mixture weights as constants and thus still independently from all covariates. In the context of reference intervals, this effectively means that the proportion of pathological cases is the same across all ages, sex and other potentially important factors. Mixture Density Networks (MDN) [18] are an alternative approach to estimate conditional finite mixture models that has become increasingly popular over the last decade. While also using the (negative) log-likelihood function of the mixture as its loss function, the parameters are estimated using the framework of artificial neural networks [19, 20]. In contrast to the EM-algorithm, defining the mixture weights as additional output nodes then allows the estimation of conditional mixture weights depending on the input.

This article investigates the performance and applicability of MDN’s for the indirect estimation of continuous reference intervals. For this purpose, we use different implementations of a basic MDN and compare it to the GAMLSS-EM approach in a series of simulation studies with varying setups to reflect different challenges. In addition, we apply all approaches to a large dataset of hemoglobin concentration measurements [21].

## Methods

The general concept underlying the algorithms applied and evaluated in this article are finite mixture models [22–24]. These models are based on the idea that a single distribution function is not able to appropriately describe the data of interest. This can, for example, be the result of unobserved heterogeneity where (categorical) variables that correlate with the outcome of interest are not observed and it is therefore not possible to directly account for these differences in the estimated model. As already explained in the introduction, this is exactly the case for the unlabelled health status of individual measurements in laboratory databases, i.e. whether or not they are to be considered pathological with regard to a specific analyte. Given the number of components *M* (roughly translating to the categories of the unobserved variable), a weighted sum of $m=1,\dots ,M$ probability density functions $f_{m}(y^{(i)},\varvec{\theta }_m)$ with corresponding parameter vector $\varvec{\theta }_m$ can then be used to construct a finite mixture distribution

$$\begin{aligned} f\big (y^{(i)}\big )=\sum _{{m=1}}^{M}\alpha _{m}f_{m}\big (y^{(i)},\varvec{\theta }_m\big ), \end{aligned}$$

where the mixture component weights $\alpha _{m}$ determine the proportion to which each component contributes to the overall model. If $\alpha _{m}>0~\forall ~m=1,\dots ,M$ and $\sum _{{m=1}}^{M}\alpha _{m}=1$, $f(x^{(i)})$ is a convex combination of all $f_{m}(x^{(i)},\varvec{\theta }_m)$ and again a probability density function.

*Latent class regression / cluster-wise regression* models [25, 26] extend the basic mixture formula presented above by allowing one or more of these parameters to be functions of the observed covariate vector ${\varvec{x}}^{(i)}$. Although most applications focus only on the location parameter of the components, a general formula of the resulting conditional mixture model is given by

$$\begin{aligned} f\big (y^{(i)}\big |{\varvec{x}}^{(i)}\big )=\sum _{{m=1}}^{M}\alpha _{m}\big ({\varvec{x}}^{(i)}\big )f_{m}\Big (y^{(i)},\varvec{\theta }_{m}\big ({\varvec{x}}^{(i)}\big )\Big ). \end{aligned}$$

Both the component weights as well as the component-specific distribution parameters of the mixture models are usually unknown and estimated from the data. For this purpose, a common strategy is the use of expectation-maximization (EM) [17]. The framework is also applicable in latent class regression settings [27] and has further already been evaluated in the context of reference interval estimation [15]. However, EM-algorithms usually estimate the mixture weights independent from covariates, i.e. all $\alpha _{m}\big ({\varvec{x}}^{(i)}\big )$ reduce to $\alpha _{m}$. As stated in the introduction, artificial neural networks are a viable alternative for estimating the unknown parameters of a mixture model. These Mixture Density Networks (MDN) [18, 28] are not restricted to independent mixture weights, but usually involve a higher number of model parameters to be estimated and are hence more demanding regarding sample sizes.

While there are other approaches for latent class regression available, this article focuses on the evaluation of different applications of MDNs for reference interval estimation in comparison with the previously proposed EM-algorithm. The remainder of this Section hence provides more details on the implementation of the different algorithms. All analyses are conducted using the free software environment for statistical computing $\texttt {R}$ [29] and the corresponding code is provided in the Additional files together with a short instruction on how to recreate the simulated scenarios.

### Mixture density network

The general structure of the applied artificial neural networks used in the simulation study is closely related to the original MDN proposed by Bishop [18]. The loss function to be minimized is simply the negative log-likelihood of the mixture, i.e.

$$\begin{aligned} -\ln {\mathcal {L}} = -\ln \left( \prod _{i=1}^n \sum _{m=1}^M {\hat{\alpha }}_{m}\big ({\varvec{x}}^{(i)}\big )f_{m}\Big (y^{(i)},\hat{\varvec{\theta }}_{m}\big ({\varvec{x}}^{(i)}\big )\Big )\right) \end{aligned}$$

with the hat above the unknown parameters indicating them being estimated from the data. For this purpose, the network feeds the input to a single hidden layer with $\tanh$ activation functions. If the mixture is using the same probability density function with *K* parameters for all *M* components, the output layer for a univariate outcome variable $y^{(i)}$ consists of $(K+1)M$ output neurons. As this article focuses on Gaussian components, $\varvec{\theta }_{m}\left( {\varvec{x}}^{(i)}\right) {:}{=}\left( {\mu }_{m}\left( {\varvec{x}}^{(i)}\right) ,{\sigma }_{m}\left( {\varvec{x}}^{(i)}\right) \right)$ and *K* hence equals two.

The model weights are estimated via two different methods. First, we use the BFGS algorithm [30] in the standard distribution of R [31] together with the gradients provided by Bishop [28] and report the results after convergence. As the most striking advantage of using artificial neural networks over the EM-algorithm in this comparison is the ability to estimate conditional mixture weights $\hat{\varvec{\alpha }}\big ({\varvec{x}}^{(i)}\big )$, we run the algorithm once with the output fully connected to the hidden layer and again without connecting the units reserved for the mixture weights. As the latter of course results in constant mixture weights independent from the input, the model serves as middle ground between EM and MDN. In the remainder of the article, we refer to the independent and dependent setup as $\text {BFGS}_{\alpha }$ and $\text {BFGS}_{\alpha (x)}$, respectively. The second estimation strategy uses the $\texttt {R}$ interface to $\texttt {Keras}$ and $\texttt {TensorFlow (Probability)}$ [32, 33]. This results in a small difference regarding the activation function for the standard deviations $\varvec{\sigma }\left( {\varvec{x}}^{(i)}\right)$, as the softplus function is used as default instead of the “plain” exponential function. In addition, the gradients are calculated via automatic differentiation and the popular ADAM optimizer [34] is used and initialized with random glorot uniform starting weights [35]. Finally, $20\%$ of the data are reserved for a validation set to employ early stopping with patience parameter set to 20.

### Expectation-maximization

The implemented EM-algorithm is basically identical to the approach described in Hepp et al. [15] (also provided in the Additional files) and can be considered as the current benchmark for conditional mixture modeling in the field of reference interval estimation. The model parameters of the mixture components are estimated via the maximum likelihood approach implemented in the $\texttt {gamlss}$-package for generalized additive models for location scale and shape [36]. However, for the sake of improving the comparability we use $\tanh$ bases to estimate the non-linear terms for all components instead of the “regular” cubic B-splines commonly used in the application of generalized additive models to match the activation functions of the hidden layer in the MDN. For a single continuous covariate ${\varvec{x}}$ the *b*-th of $1,\dots ,B$ bases is calculated as

$$\begin{aligned} \tanh \left( \alpha _b+\beta _b\cdot {\varvec{x}}\right) , \end{aligned}$$

where

$$\begin{aligned} \alpha _b=\frac{-B\left( \min ({\varvec{x}})+\frac{(b-1)}{B-1}(\max ({\varvec{x}})-\min ({\varvec{x}}))\right) }{\max ({\varvec{x}})-\min ({\varvec{x}})} \end{aligned}$$

and

$$\begin{aligned} \beta _b=\frac{B}{\max ({\varvec{x}})-\min ({\varvec{x}})} \end{aligned}$$

A Figure with examples for three different choices of *B* is provided in the Additional file 4.

As mentioned earlier, the choice of appropriate starting weights is a very important prerequisite for the algorithm to provide reasonable results. As a consequence, we use the initialization strategy proposed in the original article based on the cumulative distribution function of the Gaussian distribution with parameters estimated via the ‘naive’ maximum-likelihood fit on the data.

## Results

In order to investigate and compare the different approaches we first adopted the simulation study used in Hepp et al. [15] as baseline setting. However, since a key difference between the applied algorithms is their ability to account for the effects of covariates on the component weights, we extended the setting to highlight the performance in scenarios where the proportions of the components in fact vary for different values of a covariate. In addition, we use data from the PEDREF reference interval initiative [21] to assess performance in a real-world example.

### Simulated data

The data-generating process for this simulation study is a Gaussian mixture with two components both depending on a single predictor variable $x^{(i)}\sim {\mathcal {U}}(0,1)$. In order to mimic the conditions likely to be found in laboratory databases, the components are further positioned above each other intended to represent the “physiological” reference distribution and the “pathological” values. While the shape of the effect on location and scale (i.e. mean and standard deviation for Gaussian distributions, respectively) is always the same, a total of four different scenarios are examined, which differ in terms of sample size and the relationship between $x^{(i)}$ and the weights of the mixture components (independent/dependent). A detailed description of the process is provided in the Additional files.

**Fig. 1:**
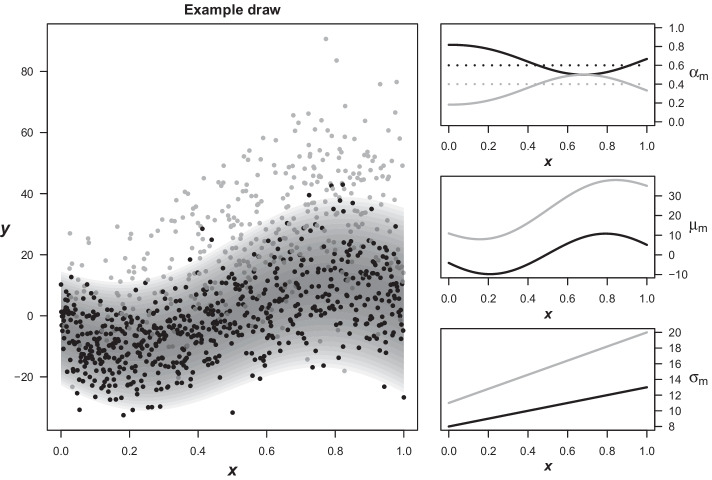
Simulation example. Left: Example draw of 5000 points from the data-generating process. Samples from the “main” component are colored black and backgrounded with an area depicting the density of their parent distribution. Grey points are sampled from the “pathologic” component. Right: True parameters of the data-generating process. Regarding the mixture component weights, the dependent and independent scenarios are depicted by solid and dashed lines, respectively

Each setting is repeated for $R=100$ simulation runs, resulting in the evaluation of a total of 400 datasets. The left panel in Fig. 1 shows an example draw of $n=5000$ observations with the true density of the “main” distribution highlighted by the shaded area in the background and samples from the pathologic distribution colored grey. Finally, the solid lines show the location parameter of both components. For the sake of improving the comparability further, we used the same number of basis functions $B=5$ for the estimation of the distribution parameters in all applied models, which translates into a single hidden layer with five nodes for the MDNs. The models fitted via the EM-framework use five tanh-bases as described at the end of the Methods Section. Both input and output variables were standardized by substracting the sample mean and dividing by the sample standard deviation before model fitting.

Since the labels of the estimated components are completely interchangeable, the results of the simulations are first unified by presuming some a priori knowledge about the approximate location of the healthy samples, which is quite reasonable in the actual use case of reference distributions. For this purpose, the component for which $\sum _{i=1}^n\mu (x^{(i)})$ is smaller is mainly positioned below the other and is hence identified as the main component with $m{:}{=}1$. To compare the performance of the algorithms, we calculated the squared error for the mixture component weights as well as the location and scale parameters of the main component on the original scale of input and output. However, the first examination of the results showed that the errors of some or all of the applied MDN algorithms “exploded” in a considerable part of the simulation runs. This can be traced back to a problem also encountered by the EM algorithm if it is initialized with random observation weights. To be specific, at some point during model fitting the estimated location parameters may eventually cross each other and the optimizer converges to a local minimum of the loss function, resulting in parts of the estimated components switching places.

**Fig. 2:**
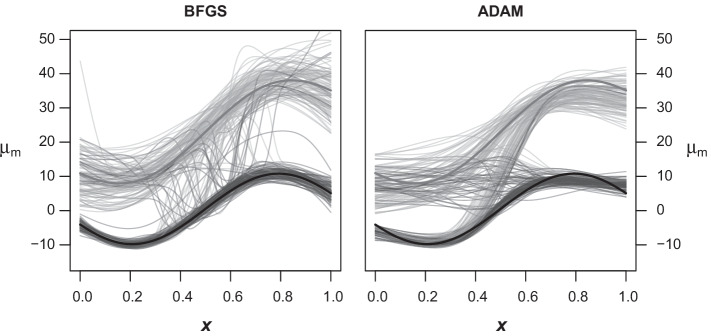
Component switching. Identifiability problems promoted by unfavorable random initial model weights illustrated by the estimated location parameters alternating between the true component means. Dark grey lines represent the main component, with the slightly more wide solid lines representing the true location parameter of the data-generating process. Left: Results from the BFGS optimizer on the fully connected MDN. Right: Results of the ADAM optimizer with early stopping

Figure 2 shows both component locations estimated via the BFGS and ADAM optimizers for all 100 simulation runs in the setting with mixture weights depending on the input variable. Considering the large jumps of the estimated parameters between the two actual components, the huge errors in the affected runs are not surprising. While the overall model may even describe the data reasonably well, the individual parameters themselves of course deviate strongly from where they should be once switching places. The main problem, however, is that the reference intervals derived from the corresponding components would clearly be invalid because they switch between describing the distribution of physiological and pathological samples for different ranges of the covariate. Table 1 provides an overview on the frequency of location crossing for each algorithm and simulation setup. With respect to the MDN models, all randomly initialized algorithms exhibit component switching in at least 15% up to 53% of the corresponding runs.

**Table 1:** Number of runs out of 100 with location crossing for each algorithm

Setting		EM	BFGS_*α*_		BFGS_*α*(*x*)_		ADAM	
n	*α*		Random	Custom	Random	Custom	Random	Custom
10000	Dep	0	37	16	29	16	53	1
	Indep	0	27	14	18	8	39	0
5000	Dep	0	32	19	36	14	48	4
	Indep	0	23	17	15	11	44	0

While the EM-algorithm seems to be completely unaffected, it should again be noted that the applied version uses custom initial observation weights in order to prevent exactly this problem from occuring. As a consequence, we aimed to improve the performance of the MDNs in a similar way by using a partially customized set of starting weights that already positions the components above each other. We tried to keep this approach as simple as possible by using ordinary least squares estimation on $B=5$ tanh-bases generated as described in the Methods. Then, the 10% and 90% quantiles of the residuals are calculated and added to the intercept. The coefficients of the resulting two shifted versions of the original OLS estimation are used as initial weights for the network output regarding the location parameter of the two components $\varvec{\mu }(x^{(i)})$, while the coefficients for the tanh-bases are used as initial weights for the hidden units. All remaining weights from the hidden layer to the scale and mixture weight outputs remain randomly initialized. An detailed explanation of the procedure is provided in the Additional files with an illustration based on the examplary data in Fig. 1.

Using these adjusted starting weights clearly improved the performance by reducing the number of runs with component switching for MDNs estimated via BFGS, but even more so for those estimated via ADAM even though the latter performed worse using strictly random initial weights (Table 1). Moreover, a minor proportion of the BFGS algorithms failed to identify the mixture and tried to fit the data with a single component. As a consequence, we also removed these runs to base the comparison of the estimated parameters on situations where the main component has been identified correctly (see the Additional file 4 for a Table providing the number of affected runs per algorithm).

**Fig. 3:**
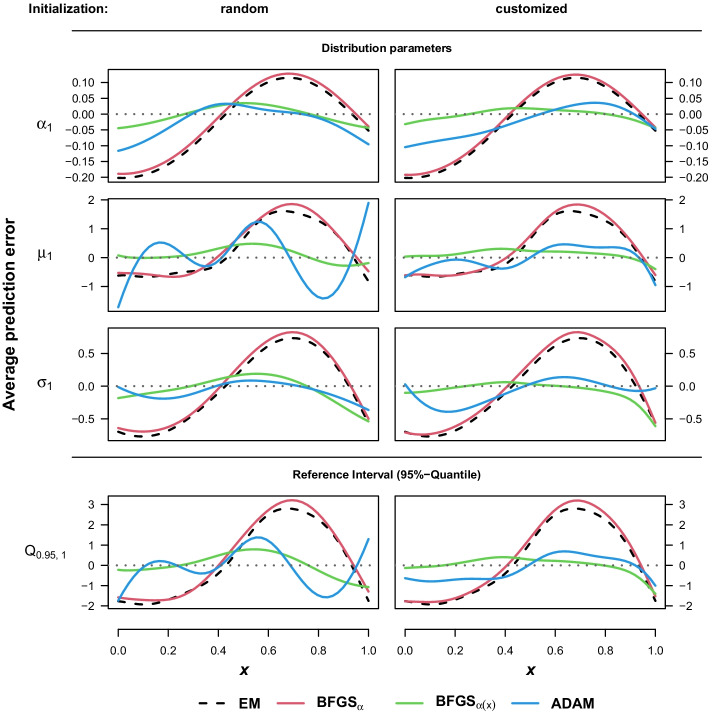
Average prediction error (dependent setting). Average prediction error over the full range of the input variable for the parameters of the first component (top three rows) and the 95% quantile (bottom row). Predictions are based on the results without location crossing from the simulation setup with mixture weights depending on the input and sample size $n=10000$

Figure 3 illustrates the average prediction error over the full range of the input variable for the parameters together with the 95%-quantile of the first component that could serve as reference threshold in an actual application setting. The predictions are based on the model estimates resulting from the simulation setup with mixture weights non-linearly depending on the input and sample size $n=10000$. The error is then obtained from the deviation to the corresponding values of the data-generating process. Unsurprisingly, both algorithms that assume constant mixture weights show higher errors with respect to $\alpha _1\big (x^{(i)}\big )$ itself, with no obvious differences regarding the initialization strategy. More interesting, however, is that this assumption directly affects the accuracy of the predictions of the distribution parameters as well, with the BFGS optimizer on the MDN with constant mixture weights performing worst. Moreover, although runs with location crossing have been excluded, the customized starting weights seem to improve the performance of the ADAM optimizer regarding the estimation of $\mu _1\big (x^{(i)}\big )$, but not necessarily $\sigma _1\big (x^{(i)}\big )$.

**Fig. 4:**
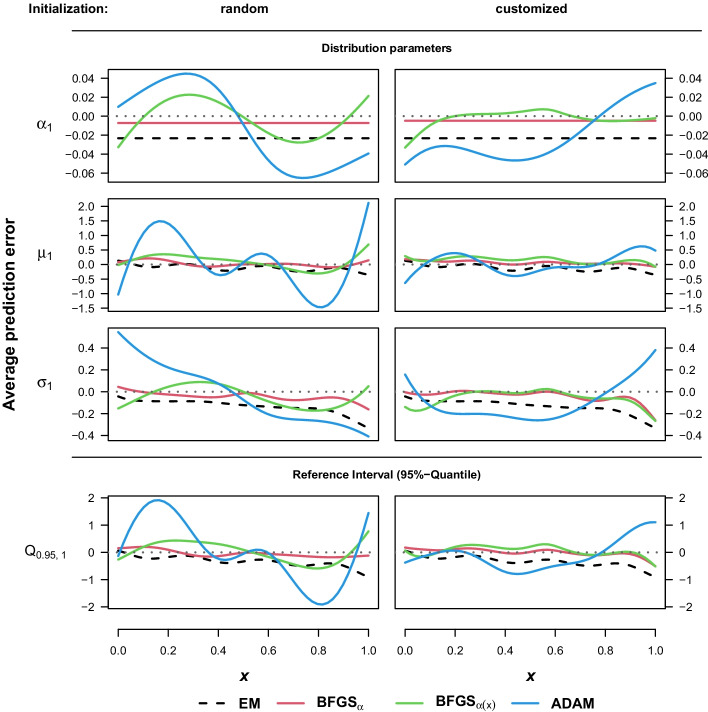
Average prediction error (independent setting). Average prediction error over the full range of the input variable for the parameters of the first component (top three rows) and the 95% quantile (bottom row). Predictions are based on the results without location crossing from the simulation setup with mixture weights independent from the input and sample size $n=10000$

As should be expected, both EM and the BFGS variant with the corresponding model assumption perform better in the simulated data settings with constant mixture weights, the latter being slightly superior (see Fig. 4). While the average predictions from the ADAM optimizer are arguably the worst in these settings, the full MDN with BFGS performs actually quite well despite the misspecification of the mixture weights, especially if the customized starting weights are used. Then it even outperforms the EM algorithm in all other predictions, i.e. location, scale and the 95%-quantile of the main component.

**Table 2:** Average integrated squared error as the average difference between predicted value and data-generating process

Setting		EM	BFGS_*α*_		BFGS_*α*(*x*)_		ADAM	
*α*	n		Random	Custom	Random	Custom	Random	Custom
Average $\text {ISE}({\hat{\alpha }}_1)$								
Dep.	10000	0.0139	**0.0135**	0.0137	**0.0045**	0.0057	0.0067	0.0078
	5000	0.0149	0.0145	**0.0143**	**0.0073**	0.0099	0.0092	0.0125
Indep.	10000	0.0021	**0.0019**	**0.0019**	**0.0049**	0.0065	**0.0049**	0.0071
	5000	**0.0032**	0.0042	0.0037	**0.0079**	0.0109	0.0087	0.0096
Average $\text {ISE}({\hat{\mu }}_1)$								
Dep.	10000	**1.2292**	1.4308	1.4921	**0.6809**	0.8533	1.2838	0.8794
	5000	**1.6960**	1.8426	2.1324	**1.3833**	1.8223	1.9156	1.7716
Indep.	10000	**0.3875**	0.4078	0.4649	**0.6731**	0.9459	1.2989	0.8172
	5000	**0.8185**	0.8941	0.9648	**1.3963**	1.9779	2.0296	1.5842
Average $\text {ISE}({\hat{\sigma }}_1)$								
Dep.	10000	0.4588	**0.4583**	0.5003	0.2780	0.3837	**0.2672**	0.3645
	5000	0.6584	**0.6347**	0.7312	0.4800	0.7890	**0.3657**	0.6859
Indep.	10000	0.1992	**0.1813**	0.2173	**0.2741**	0.4116	0.2797	0.3574
	5000	0.3370	**0.3286**	0.4086	0.4686	0.7624	**0.4462**	0.5760
Average $\text {ISE}({\hat{Q}}_{0.95,1})$								
Dep.	10000	**4.4896**	4.9129	5.1704	**2.4881**	3.3152	2.8388	3.0161
	5000	**6.1319**	6.3538	7.2327	4.6173	6.8189	**4.0797**	6.2077
Indep.	10000	1.5022	**1.4790**	1.6823	**2.4710**	3.5811	2.9686	2.8411
	5000	**2.7713**	2.9091	3.2766	**4.5883**	7.0449	4.9499	5.2115

Bold numbers denote the smallest value comparing EM to $\text {BFGS}_{\alpha }$ and $\text {BFGS}_{\alpha (x)}$ to ADAM in each setting. Runs with location crossing or failing to identify two components are excluded for the corresponding algorithms

In order to provide a summarized view of the results in all settings and variations, Table 2 provides the average integrated squared error $\text {ISE}(\hat{\theta })=\int _0^1 \big (\hat{\theta }(x)-{\theta }(x)\big )^2~dx$ for the same parameters as depicted in Figs. 3 and 4 . While the general findings taken from these Figures are also evident in the average ISEs, the models with customized initial weights seem to perform slightly worse when compared to their randomly initialized counterparts. This is also true for the $\text {BFGS}_{\alpha (x)}$ estimates, despite the improvement in the average prediction error noted in the independent setting in Fig. 4. The important exception are the average $\text {ISE}({\hat{\mu }}_1)$ of the ADAM optimizer, which clearly benefits from the advantageous starting position provided by the customized weights. It should be reminded, however, that the results are again based only on the runs of the respective algorithms with both components properly identified, i.e. without location crossing and $\frac{1}{n}\sum _{i=1}^{n}\alpha _m(x^{(i)})\ge 0.05$ for all *m*. Regarding the models assuming constant $\alpha _m$, there might be a small tendency in favor of the EM algorithm especially in the settings with the number of available samples at $n=5000$ which could be explained by the fact that it requires fewer unknown coefficients to be estimated. In the comparison between the fully connected models, ADAM with early stopping surpasses BFGS optimization only with respect to the estimation of the scale parameter $\sigma _1\big (x^{(i)}\big )$. As mentioned in Section , however, the default output layer for mixtures of Gaussian distributions in $\texttt {TensorFlow Probability}$ applied in this simulation study uses the softplus activation function for the standard deviation. Therefore, these results could also indicate a possible advantage over the standard exponential function.

### Hemoglobin concentration

In addition to the simulation settings evaluated above, we apply all candidate algorithms for the estimation of pediatric hemoglobin reference intervals. The available laboratory tests were performed in the context of patient care in the Department of Pediatrics and Adolescence at the University Hospital Erlangen, Germany as part of the PEDREF reference interval initiative [21]. For this comparison, we used test results regarding hemoglobin concentration of girls aged between one and eighteen years, irrespective of health status or specialty unit, including intensive care and oncology units. After removing samples taken at subsequent visits of the same individual, a total of $n = 60423$ test results were availablefor the analysis.

In order to assess the stability of the estimates, we ran each algorithm 100 times and used a different set of initial values in the corresponding random component of the starting weights for each repetition. Interestingly, the problems discussed in the previous section simulation setup, i.e. location crossing and failure to identify more than a single component in the data, also occured in a few runs despite the large sample size. However, comparing the loss of all repetitions revealed relatively poor negative log-likelihood values for the affected models.

**Fig. 5:**
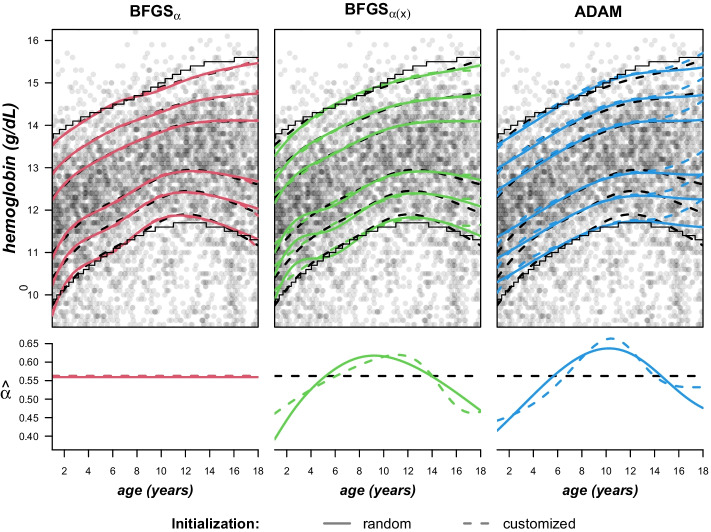
Reference intervals for hemoglobin concentration. Top row: Hemoglobin concentration of girls with estimated quantiles $Q_p$ for the healthy component where $p \in (0.025,0.1,0.25,0.75,0.9,0.975)$. Displayed results show the solutions with minimum loss after 100 repeated initializations. Dashed black lines represent the EM-algorithm, the slightly finer solid lines discrete results from an alternative approach not based on latent class regression. Bottom row: Estimated mixture component weight of the healthy component

Figure 5 shows the $2.5\%,10\%,25\%,75\%,90\%$ and $97.5\%$ quantiles of the main components for all applied MDN algorithms together with the estimation of the corresponding mixture component weight below. The depicted estimates are derived from the runs with minimum loss. For ease of differentiation, the three competing approaches are depicted side by side in separate plots. For reference, the results of the EM algorithm are shown in all three columns along with the discrete results (only 2.5% and 97.5%) from an alternative approach which requires splitting of the dataset.

On a first note, all methods in the latent class regression framework, i.e. MDNs as well as EM, provide slightly more conservative solutions than the alternative discrete method in the sense that the proposed intervals are narrower. With respect to the more interesting 2.5%-quantile (low hemoglobin concentration is considered pathologic), the MDN results for $\text {BFGS}_{\alpha }$ and ADAM are closer to these stepwise estimates in the middle of the observed age range, whereas the models with constant component weights are closer on the edges. The comparison of EM and $\text {BFGS}_{\alpha }$ yields rather identical results, especially regarding the estimated component weights. In this regard, both $\text {BFGS}_{\alpha (x)}$ and ADAM show an increasing proportion of healthy samples until about 10 or 11 years of age and a decreasing proportion thereafter. Moreover, the quantiles of the estimated distributions show somewhat larger differences relative to the component determined by the EM algorithm. Additional differences occur especially at the peripery of the age variable. To be specific, the estimates of the randomly initialized $\text {BFGS}_{\alpha (x)}$ model exhibit slightly increased variability especially at a young age, while the opposite can be observed for the result of the ADAM optimizer. The latter is probably the effect of early stopping, which may regularize the estimation a little to strongly in this case. The strongest deviation from the EM results can be observed for ADAM with the (partially) customized starting weights, which is the only model identifying a clear increase of all quantiles beginning at the age of fourteen.

**Fig. 6:**
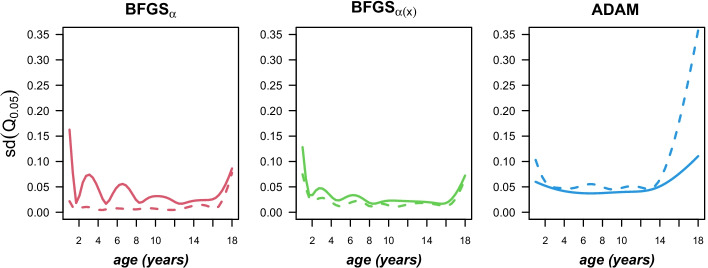
Variability of estimated quantiles. Standard deviation of the $5\%$ quantiles derived from 100 runs with varying random component in the corresponding intial weights

In this context, customizing the starting weights interestingly has actually the opposite effect on the variance of the estimated quantiles obtained from the two different optimization strategies. Figure 6 illustrates this using the estimated $5\%$-quantiles, where the stabilizing propertiy of the custom weights on the standard deviation of the BFGS output is clearly visible, whereas the opposite is true for ADAM. In fact, the standard deviation rises rather steeply in the area where the corresponding quantiles in Fig. 5 deviate heavily from the other algorithms.

Revisiting the model results presented earlier, both solutions from the ADAM optimizer actually result in the highest values with respect to the loss, while the smallest loss is achieved by the purely randomly initialized BFGS algorithm with constant component weights. While this may seem at first to be a contradiction to the results of the simulation study, it should be reminded that the loss is calculated from all components in a mixture and is therefore not necessarily a good indicator of the accuracy regarding the identification of a specific component.

## Discussion

This article examines the suitability of artificial neural networks in the form of mixture density networks (MDN) in a latent class distributional regression framework for the estimation of indirect reference intervals to support in vitro diagnostics. In contrast to most established methods, the proposed approach enables the estimation of physiological and pathological components from unlabeled data while simultaneously adjusting for covariates such as age. Moreover, the framework is not restricted to a single input but able to incorporate multiple continuous and/or categorical variables and thereby able to capture potential interaction effects or other interesting dependecy patterns. Previous estimation strategies based on the EM-algorithm already provided promising results, but were not able to model potential patterns regarding the relationship between the component weights of the mixture (i.e. the proportion of healthy samples) and the input variable(s).

As a varying proportion of healthy and pathological samples with respect to important covariates such as age is actually a quite reasonable assumption for laboratory databases, neglecting these potential dependencies clearly raises the risk of biased reference intervals. This is clearly illustrated by the results of the simulation study, which demonstrate that if such a dependence of the mixture components on the input is present in the data, neglecting it will eventually lead to biased estimates of all other distribution parameters as well. All variants of the fully connected MDNs clearly and consistently outperform the reduced (and thus misspecified) models with constant component weights in the fully conditional simulation settings. Although the opposite is true in the rather theoretical case of independent settings, the differences are less pronounced and more likely to be due to their lower accuracy than systematic bias.

While the proposed fully connected mixture density networks should be preferred over the EM-algorithm for this reason, our results also indicate that both initialization and optimization strategy affect the results of the network and must be carefully considered. Like many applications of mixture models and latent class regression in particular, MDNs also struggle with identification problems. Both evaluated optimization strategies often moved towards solutions where the estimated component locations switched places somewhere over the range of the input variable and were then stuck in these local optima. By using partially customized initial weights, this problem could often be avoided, as the algorithm then starts in a relatively favorable position. However, implementing these weights requires reasonable assumptions about the true location of the components and is probably a little less straightforward than setting up customized observation weights for the EM algorithm. On the other hand, initializing the model multiple times—as is common practice in the application of most artificial neural networks – might generally help to mitigate this problem as seen in the hemoglobin data example.

Considering only the solutions with both components properly identified, the BFGS algorithm performed slightly better than ADAM with early stopping. It should be noted, however, that the setup for all applied networks consisted of a single hidden layer with only five hidden units. As a consequence, severely overfitting the data by running the BFGS until convergence is not really possible and might actually be more prominent if more hidden units are added. A small indication of this can possibly be observed in the application on the hemoglobin data, where the BFGS solutions are slightly more dynamic in comparison. Early stopping as implemented together with the ADAM optimizer, on the other hand, may regularize the model too strongly in this case. Additional experiments with different setups and strategies, e.g. $L_2$-penalties on the weights from the input to the hidden layer, are therefore required. Moreover, we limited the simulation study to settings with two Gaussian components and a single input variable. While this evaluation is already very helpful with respect to the hemoglobin concentration example, these assumptions are not meaningful for a large variety of other analytes. Further studies with asymmetric distributions and multiple pathologic components have to be conducted to expand the scope of applications to a wider range of analytes. However, it is expected that the resulting increase in the number of output parameters will place greater demands on sample size and potentially further complicate the correct identifiability of the separate components, calling for more sophisticated solutions than the cutomized starting weights.

## Conclusion

In this first evaluation of mixture density networks in the context of indirect reference interval estimation, our results demonstrate the ability of the applied algorithms to correctly identify the latent components in unlabelled data. By explicitly modelling the dependency structure of the component weights, i.e. the proportion of physiological/pathological samples in the database, the fully connected MDNs avert biased distributions and their corresponding quantiles required for the creation of reference intervals. Using a set of partially customized starting weights further greatly reduces the occurence of identifiability problems due to the high flexibility and relatively large number of estimated parameters in this framework. Further investigations regarding appropriate regularization strategies are desired to determine the best practice and provide appropriate guidelines to extract the most accurate and reliable intervals. Moreover, settings with multiple output and/or input variables may eventually help to provide a deeper understanding of the relationship between different analytes and their dependence on a patient’s individual characteristics.

## Supplementary Information

**Additional file 1. **R-script containing the data-generating function for the simulation study.

**Additional file 2. **R-script containing the functions to estimate the model via expectation-maximization.

**Additional file 3. **Main R-script used to compute results and produce figures.

**Additional file 4. **Appendix/Supplementary Figures.

**Additional file 5. **R-script containing the functions to estimate the model via BFGS.

**Additional file 6. **R-script containing the simulation study.

**Additional file 7. **R-script containing utility functions used by some of the other scripts.

## Data Availability

The R-scripts used to both generate and analyze the datasets supporting the conclusions are provided as Supplementary Information files. Hemoglobin data is provided by the PEDREF reference interval initiative (https://www.pedref.org/index.html) and available there upon reasonable request.

## Abbreviations

MDN: Mixture density network
GAMLSS: Generalized additive model for location, scale and shape
EM: Expectation-maximization (algorithm)
BFGS: Broyden–Fletcher–Goldfarb–Shanno (algorithm)
ADAM: Adaptive moment estimation
PEDREF: Pediatric reference interval initiative
ISE: Integrated squared error
