# Supplementary material for: Mixture density networks for the indirect estimation of reference intervals
Source: BMC Bioinformatics. 2022 Jul 29;23:307. doi: 10.1186/s12859-022-04846-0 (PMC9336034; doi:10.1186/s12859-022-04846-0)
Supplement: Supplementary file 4 — Additional file 4. Appendix/Supplementary Figures. [file 12859_2022_4846_MOESM4_ESM.pdf]

Additional Files

A1 Expectation-maximization for latent class distributional regression

Algorithm 1

Initialization

1. Set the iteration counter $t = 0$ and specify the total number of components M
2. Provide starting weights $w_m^{(i,t)}$ for all $i = 1, \dots, n$ observations in all $m = 1, \dots, M$ components.
3. For $m = 1, \dots, M$:
   - Fit a GAMLSS to the data weighted by $w_m^{(i,t)}$ to estimate all K distribution parameters
   $$\hat{\boldsymbol{\theta}}_m^{(i,t)} = \left(\hat{\theta}_1^{(t)}(\boldsymbol{x}^{(i)}), \dots, \hat{\theta}_K^{(t)}(\boldsymbol{x}^{(i)})\right)$$
   using the family-specific default initial values.
   - Compute the mixture weights
   $$\alpha_m^{(t)} = \frac{1}{n} \sum_{i=1}^{n} w_m^{(i,t)}$$
4. Compute the log-likelihood
$$\ln \mathcal{L}^{(t)} = \ln \left(\prod_{i=1}^{n} \sum_{m=1}^{M} \alpha_m^{(t)} f\left(y^{(i)}, \hat{\boldsymbol{\theta}}_m^{(i,t)}\right) \right).$$

Iterations

1. Set $t := t + 1$.
2. For $m = 1, \dots, M$: Update the weights
$$w_m^{(i,t)} = \frac{\alpha_m^{(t-1)} f\left(y^{(i)}, \hat{\boldsymbol{\theta}}_m^{(i,t-1)}\right)}{\sum_{m=1}^{M} \alpha_m^{(t-1)} f\left(y^{(i)}, \hat{\boldsymbol{\theta}}_m^{(i,t-1)}\right)}$$
3. For $m = 1, \dots, M$:
   - Fit a GAMLSS to the data weighted by $w_m^{(i,t)}$ to estimate all K distribution parameters using the estimates from the previous iteration $\hat{\boldsymbol{\theta}}_m^{(i,t-1)}$ as initial values.
   - Compute the mixture weights
   $$\alpha_m^{(t)} = \frac{1}{n} \sum_{i=1}^{n} w_m^{(i,t)}$$
4. Compute the log-likelihood $\ln \mathcal{L}^{(t)}$
5. Repeat steps 1-4 until $\ln \mathcal{L}^{(t)} - \ln \mathcal{L}^{(t-1)} \leq \epsilon$, where ϵ is the desired convergence threshold.

A2 Examples of the custom tanh-basis functions

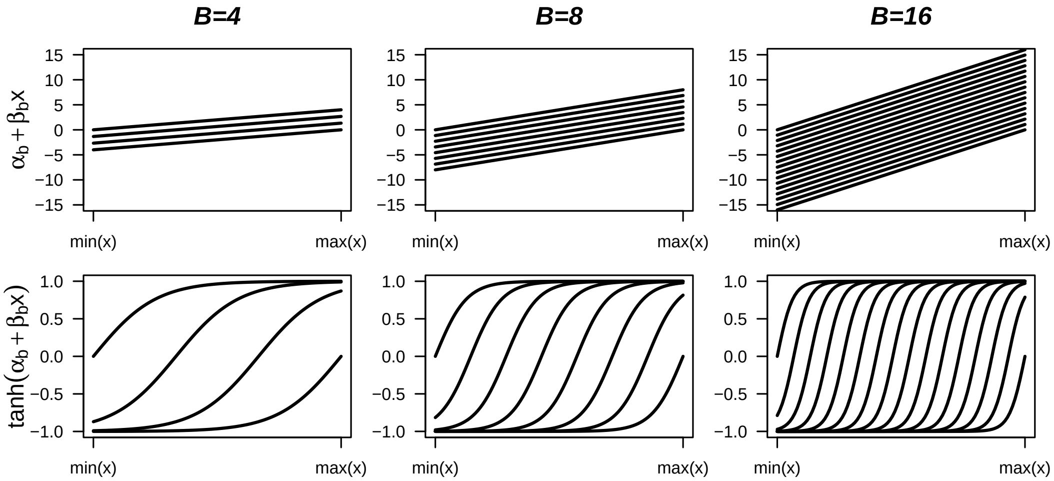

Figure 1: Examples of the custom tanh-basis functions with different number of bases B

A3 Simulation setup

The responses are sampled from the two-component Gaussian mixture

$$y^{(i)} \sim \alpha_1(x^{(i)})\ \mathcal{N}\left(\mu_1(x^{(i)}), \sigma_1(x^{(i)})^2\right) + \alpha_2(x^{(i)})\ \mathcal{N}\left(\mu_2(x^{(i)}), \sigma_2(x^{(i)})^2\right)$$

with all $x^{(i)}$ drawn form the standard uniform distribution $\mathcal{U}(0,1)$. The first component is considered to be the distribution of interest, i.e. the distribution attributed to the "healthy" part of the sample with

$$\mu_1(x^{(i)}) = x^{(i)} + 10\sin\left((x^{(i)} - 0.5)\sqrt{3}\pi\right) \qquad \text{and} \qquad \sigma_1(x^{(i)}) = \exp\left(8 + 5x^{(i)}\right).$$

The second component is parameterized by

$$\mu_2(x^{(i)}) = 15 + 15x^{(i)} + 10\sin\left((x^{(i)} - 0.5)\sqrt{3}\pi\right) \qquad \text{and} \qquad \sigma_2(x^{(i)}) = \exp\left(11 + 9x^{(i)}\right).$$

With respect to the mixture component weights, two scenarios are investigated:

- Independent: $\alpha_1(x^{(i)}) = 0.6$
- Non-linear: $\alpha_1(x^{(i)}) = g\Big(0.75 + 0.75\sin\left(1.5 + 1.5x^{(i)}\pi\right)\Big)$

Here, $g()$ is the standard logistic function and $\alpha_2(x^{(i)}) = 1 - \alpha_1(x^{(i)})$ for both settings.

A4 Custom initial weights for the Mixture Density Networks

The custom inital weights used in the corresponding Mixture Density Networks are determined as follows:

The b-th activated node in the hidden layer connected to a single continuous input variable $\boldsymbol{x}$ is described by

$$\boldsymbol{z}_b = \tanh\left(\alpha_b + \beta_b \boldsymbol{x}\right).$$

The custom weights from the input to the hidden layer are then

$$\alpha_b = \frac{-B\left(\min(\boldsymbol{x}) + \frac{(b-1)}{B-1}(\max(\boldsymbol{x}) - \min(\boldsymbol{x}))\right)}{\max(\boldsymbol{x}) - \min(\boldsymbol{x})} \qquad \text{and} \qquad \beta_b = \frac{B}{\max(\boldsymbol{x}) - \min(\boldsymbol{x})}$$

The m-th location output node is described by

$$\boldsymbol{\mu}_m = \gamma_m + \sum_{b=1}^{B} \delta_{mb} \boldsymbol{z}_b$$

Based on the OLS solution to the linear regression model

$$\boldsymbol{y} = \gamma^{[\text{OLS}]} + \sum_{b=1}^{B} \delta_b^{[\text{OLS}]} \boldsymbol{z}_b + \boldsymbol{\epsilon},$$

the custom weights from the hidden layer to the location nodes are

$$\gamma_m = \gamma^{[\text{OLS}]} + c_m \qquad \text{and} \qquad \delta_{mb} = \delta_b^{[\text{OLS}]},$$

where c_m is a constant shift term based on empirical quantiles of the OLS residuals. Figure 2 provides a graphical illustration of the procedure.
All other weights are randomly sampled (glorot uniform initialization).

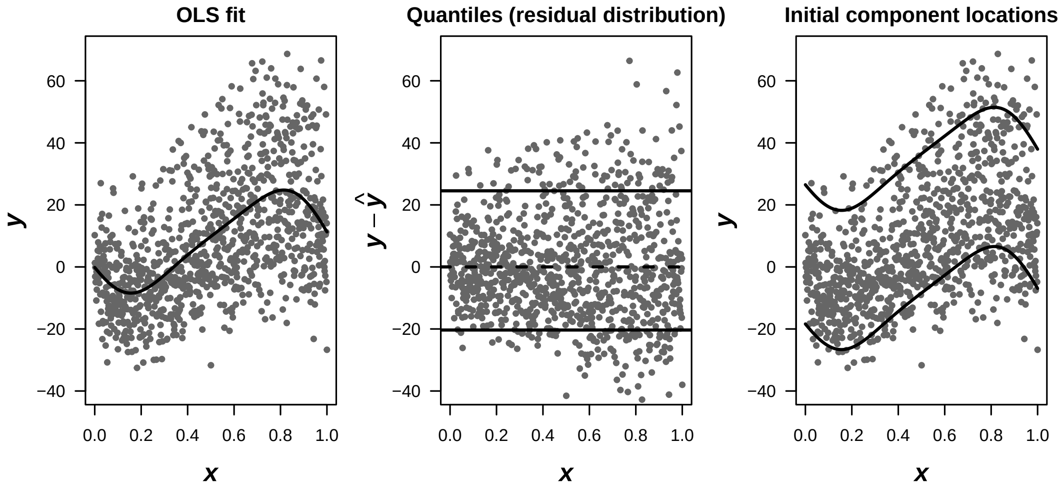

Figure 2: Schematic overview of the customized initial weights for the MDNs. Left plot shows the OLS fit on an example dataset using the tanh-bases with coefficients determined as described in the Methods section. The center plot displays the residuals from the OLS fit together with both 10% and 90% quantiles. The right plot shows these quantiles mapped back to the original data, which are then used as starting position for the location parameter of the components.

A5 Mixture not identified correctly

Table 1: Number of runs out of 100 with $n^{-1}\sum_{i=1}^{n}\alpha_1(x^{(i)})$ below 0.05 or above 0.95. Numbers in parentheses show runs in scenarios without location crossing.

Setting		EM	$BFGS_\alpha$		$BFGS_{\alpha(x)}$		ADAM	
n	α		random	custom	random	custom	random	custom
10000	dep	0	1 (0)	3 (0)	0	8 (6)	0	0
	indep	0	1 (0)	3 (0)	0	7 (3)	0	0
5000	dep	0	2 (1)	2 (0)	1 (1)	6 (3)	0	0
	indep	0	1 (1)	3 (0)	2 (1)	5 (2)	0	0
